# Diversity of multi-drug resistant *Acinetobacter baumannii* population in a major hospital in Kuwait

**DOI:** 10.3389/fmicb.2015.00743

**Published:** 2015-07-23

**Authors:** Leila Vali, Khadija Dashti, Andrés F. Opazo-Capurro, Ali A. Dashti, Khaled Al Obaid, Benjamin A. Evans

**Affiliations:** ^1^Department of Medical Laboratory Sciences, Faculty of Allied Health Sciences, Kuwait UniversitySulaibekhat, Kuwait; ^2^Laboratorio de Investigación en Agentes Antibacterianos, Departamento de Microbiología, Facultad de Ciencias Biológicas, Universidad de ConcepciónConcepción, Chile; ^3^Microbiology Department, Amiri HospitalKuwait City, Kuwait; ^4^Department of Biomedical and Forensic Sciences, Faculty of Science and Technology, Anglia Ruskin UniversityCambridge, UK

**Keywords:** *Acinetobacter baumannii*, MLST, PFGE, OXA, PER-1

## Abstract

*Acinetobacter baumannii* is one of the most important opportunistic pathogens that causes serious health care associated complications in critically ill patients. In the current study we report on the diversity of the clinical multi-drug resistant (MDR) *A. baumannii* in Kuwait by molecular characterization. One hundred *A. baumannii* were isolated from one of the largest governmental hospitals in Kuwait. Following the identification of the isolates by molecular methods, the amplified *bla*_OXA-51_-like gene product of one isolate (KO-12) recovered from blood showed the insertion of the ISAba19 at position 379 in *bla*_OXA-78_. Of the 33 MDR isolates, 28 (85%) contained *bla*_OXA-23_, 2 (6%) *bla*_OXA-24_ and 6 (18%) *bla*_PER-1_ gene. We did not detect *bla*_OXA-58_, *bla*_VIM_, *bla*_IMP_, *bla*_GES,_ *bla*_VEB_, and *bla*_NDM_ genes in any of the tested isolates. In three *bla*_PER-1_ positive isolates the genetic environment of *bla*_PER-1_ consisted of two copies of ISPa12 (tnpiA1) surrounding the *bla*_PER-1_ gene on a highly stable plasmid of ca. 140-kb. Multilocus-sequence typing (MLST) analysis of the 33 *A. baumannii* isolates identified 20 different STs, of which six (ST-607, ST-608, ST-609, ST-610, ST-611, and ST-612) were novel. Emerging STs such as ST15 (identified for the first time in the Middle East), ST78 and ST25 were also detected. The predominant clonal complex was CC2. Pulsed-field gel electrophoresis and MLST defined the MDR isolates as multi-clonal with diverse lineages. Our results lead us to believe that *A. baumannii* is diverse in clonal origins and/or is undergoing clonal expansion continuously while multiple lineages of MDR *A. baumannii* circulate in hospital ward simultaneously.

## Introduction

*Acinetobacter baumannii* is one of the most important opportunistic pathogens that causes outbreaks in hospitals and serious health care associated complications in critically ill patients. The ability to acquire multiple antibiotic resistance genes and to survive in inanimate environments are important characteristics of this nosocomial pathogen (Corbella et al., 2000). The guidelines for the antimicrobial therapy for the treatment of *Acinetobacter* infections comprise of broad spectrum cephalosporins, the β-lactamase inhibitor sulbactam, quinolones, carbapenems, amikacin, doxycycline, and minocycline. In the case of infections caused by multi-drug resistant (MDR) strains, tigecycline and colistin are recommended as the last therapeutic options either alone or in combination therapy (Talbot et al., 2006). Recently some *A. baumannii* isolates have become resistant to almost all the currently available antibiotics including colistin (Magiorakos et al., 2012). Acquisition of resistance to colistin is mostly due to modifications of the lipopolysaccharide (LPS) biosynthesis pathway (Potron et al., 2015).

In *A. baumannii* carbapenem-hydrolysing class D β-lactamases (CHDLs) are major contributors to antibiotic resistance (Poirel and Nordmann, 2006). These enzymes are predominantly expressed by transmissible *bla*_OXA_ genes including: *bla*_OXA-23_-like, *bla*_OXA-24_-like, *bla*_OXA-58_-like, *bla*_OXA-143_-like, and the intrinsic *bla*_OXA-51_-like genes typically present in *A. baumannii* (Evans and Amyes, 2014). Over expression of OXA enzymes is often associated with the presence of an insertion element upstream of the gene (Turton et al., 2006). It has been reported that if *bla*_OXA-51_ is to contribute to β-lactam resistance, insertion element ISAba1 upstream of the gene is required to act as a strong transcriptional promoter (Ruiz et al., 2007). The acquired carbapenem resistance may also be associated with the production of metallo-β-lactamases such as IMP-, VIM-, and NDM-like carbapenemases (Poirel and Nordmann, 2006).

In addition to OXA and metallo-β-lactamases, resistance to cephalosporins in *A. baumannii* can be caused by chromosomally encoded AmpC enzymes, referred to as ADC cephalosporinases, through their overexpression in the presence of ISAba1 upstream of the gene (Peleg et al., 2008; Bonnin et al., 2013), or by acquisition of extended spectrum β-lactamases (ESBLs) such as GES-, VEB-, and PER-like enzymes (Nemec et al., 2008). The *bla*_PER-1_ gene has been detected in ceftazidime-resistant *A. baumannii* strains worldwide (Peleg et al., 2008) as part of a composite-transposon called Tn1213, associated with ISPa12 (Bonnin et al., 2013). Recently we identified an *A. baumannii* isolate from Kuwait that contained a *bla*_PER-1_ gene in a composite transposon made of two copies of ISPa12 (Opazo et al., 2014) prompting an increase in the minimum inhibitory concentration (MIC) of the cephalosporins and probably contributing to the mobilization of this gene.

It is crucial to sustain the effectiveness of the limited choices of antimicrobial agents we have left. This can be achieved by means of epidemiological studies and the understanding of the evolution of endemic and epidemic strains by characterizing the MDR isolates (Hamouda et al., 2010). Most common methods for epidemiology studies used for *A. baumannii* are pulsed-field gel electrophoresis (PFGE), multilocus-sequence typing (MLST), amplified fragment length polymorphism (AFLP) analysis, and other PCR- and sequence-based methods as well as whole genome sequencing analysis (Wright et al., 2014).

The main aim of this study was to investigate the diversity of MDR *A. baumannii* in Kuwait by molecular characterization of the clinical isolates. Our results will provide a platform for future evolutionary studies in this region and beyond.

## Materials and Methods

### Bacterial Strains

One hundred *A. baumannii* isolates were collected arbitrarily from patients who were admitted to Al-Amiri Hospital from 2011 until 2012. Al-Amiri hospital is a tertiary health care provider with a 500 bed capacity. The average number of specimens processed every day in the microbiology laboratory varies from 500 to 700 which includes samples from out-patient and in-patient specialists units. A database was created based on the patients’ records containing information such as age, sex, hospital location of care and type of specimen. Specimens were processed by clinical staff members of the diagnostic microbiology laboratory using standard protocols. All isolates were identified initially by using conventional microbiological techniques based on colony morphology, biochemical analysis and by using the VITEK2 system (VITEK AMS; bioMérieux VITEK Systems Inc., Hazelwood, MO, USA). The isolates were stored in 10% skimmed milk at -70°C.

The identification was then carried out in our laboratory at species level by gyrB multiplex PCR (Higgins et al., 2007, 2010) and confirmed by sequencing of an internal portion of the rpoB gene (Gundi et al., 2009). The *bla*_OXA-51-like_ gene was also amplified, purified, and sequenced (Heritier et al., 2005).

### Susceptibility Testing

Antibiotic susceptibility testing was performed by the disk diffusion method when applicable following the Clinical and Laboratory Standards Institute [CLSI] (2012) recommendations. The bacterial suspension (the final turbidity of a 0.5 McFarland standard) was spread over the Mueller-Hinton agar homogenously and the antimicrobial disks were dispensed onto the agar plates using the disk dispenser and incubated overnight at 37°C. These antibiotic agents tested were Amikacin (30 μg), Gentamycin (10 μg), Ampicillin/sulbactam (10 μg/10 μg), Cefotaxime (30 μg), Ceftazidime (30 μg), Cefipime (30 μg), Cefoxitin (30 μg), Piperacillin (30 μg), Piperacillin/tazobactam, Ciprofloxacin (5 μg), Tigecycline (15 μg), Meropenem (10 μg), and Imipenem (10 μg). The diameter of zone of inhibition was measured (mm) for all and interpreted as recommended by the Clinical and Laboratory Standards Institute [CLSI] (2012) guidelines. There was no CLSI guideline available for tigecycline, therefore we used the breakpoints recommended for Enterobacteriaceae by European Committee on Antimicrobial Susceptibility Testing [EUCAST] (2014). The MIC was determined only for some of the antibiotics by the agar dilution method following the CLSI recommendations (**Table 1**). Colistin susceptibility was calculated by broth microdilution technique which is considered the gold standard (Hindler and Humphries, 2013). The criteria for classifying the isolates as MDR was based on recommendations of Magiorakos et al. (2012).

**Table 1:** Antimicrobial susceptibility of the MDR isolates in this study.

Antibiotic	Breakpoints (mg/L)	% S	% I	% R	MIC50 (mg/L)	MIC90 (mg/L)
Amikacin	16 ≤ I ≥ 64	52	24	24	8	128
Ampicillin/sulbactam	8/4 ≤ I ≥ 32/16	30	18	52	8	128
Piperacillin/tazobactam	16/4 ≤ I ≥ 128/4	12	9	79	128	256
Cefotaxime	8 ≤ I ≥ 64	0	0	100	128	256
Ceftazidime	8 ≤ I ≥ 32	9	12	79	128	256
Ciprofloxacin	1 ≤ I ≥ 4	18	18	64	1	4
Imipenem	4 ≤ I ≥ 16	55	0	45	8	16
Tigecycline	S ≤ 1 R > 2	100	0	0	0.25	0.25
Colistin	2 ≤ I ≥ 4	97	0	3	1	2

### Detection and Identification of *bla*_OXA_ Genes

*bla*_OXA-like_ genes including *bla*_OXA-51_, *bla*_OXA-23_, *bla*_OXA-24_, and *bla*_OXA-58_ were amplified as described previously (Woodford et al., 2006). Amplified DNA fragments were purified with Qiaquick PCR purification kits (Qiagen, Valencia, CA, USA). Both strands of the amplification products were sequenced with an ABI 3100 sequencer (Applied Biosystems Life Technologies, Thermo Fisher Scientific Inc., Grand Island, NY, USA) and were analyzed using the National Center for Biotechnology Information web site^1^.

### Detection and Identification of ESBLs and Carbapenemases

The presence of the *bla*_ADC-like_ gene (Ruiz et al., 2007) and the identification of any insertion element upstream of it, was carried out as described previously (Opazo et al., 2012). The detection of ESBLs (*bla*_PER-like_, *bla*_GES-like_, *bla*_VEB-like_, *bla*_IMP-like_, *bla*_NDM-like_, *bla*_VIM-like_) was performed by PCR according to Opazo et al. (2012).

### Characterisation of the Genetic Environment of *bla*_PER-1_

In order to investigate the immediate genetic context of the *bla*_PER-1_ gene, inverse-PCR and sequencing were performed with some modifications (Opazo et al., 2012). Briefly, the whole DNA was restricted with EcoRI endonuclease for 2 h at 37°C and then circularized using the T4 ligase for 16 h at room temperature following the manufacturer’s recommendations (New England Biolabs, Hertfordshire, UK). To determine the downstream context of *bla*_PER-1_ the circularized DNA was used as a template for a PCR using the primers invPER-F (5′-GCCGAACCAATGAAGCTATCATTGCGCAGG-3′) and invPER-R (5′-AATTTGCTCTTTTAACAGTGGGGATTGCGCTG-3′). The PCR products obtained were sequenced and analyzed. The upstream region of *bla*_PER-1_ gene was characterized as before (Opazo et al., 2012).

### Plasmid Analysis

Plasmids were extracted by the use of a commercial kit following the manufacturer’s instructions and separated by PFGE using a CHEF-DRII system (Bio-Rad, Hercules, CA, USA). The size of the only plasmid detected was calculated employing the Quantity one software v4.6.1 (Bio-Rad). The plasmid was extracted from agarose using a commercial kit (Qiagen) and used as a template for PCR with the *bla*_PER-1_ and *bla*_OXA-51_ primers to determine whether *bla*_PER-1_ is plasmid-borne and to confirm the product was free of chromosomal contamination. The contribution of the *bla*_PER-1_ gene to ceftazidime-resistance was analyzed by curing experiment at 45°C according to Opazo et al. (2014). For plasmid curing experiments a single colony was streaked onto a MacConkey agar plate every day for 30 days and incubated at 47°C. The susceptibility to ceftazidime was measured by diffusion test (Kirby–Bauer method) and the presence of *bla*_PER-1_ was detected by PCR as explained earlier.

### Multilocus-Sequence Typing

Genomic DNA was extracted by using the Wizard genomic DNA purification kit (promega, UK) followed by PCR of the seven housekeeping genes as described by the Institut Pasteur web site. The internal fragments of protein encoding housekeeping genes were amplified, sequenced, and analyzed according to Diancourt et al. (2010). The isolates were assigned to sequence types (STs) using the Institut Pasteur web site^2^.

### eBURST Analysis

eBURST analysis was conducted to investigate the evolutionary relationships and clonal complexes (CCs) within the isolates, using the software on the eBURST website^3^.

### Pulsed-Field Gel Electrophoresis

Chromosomal DNA was prepared and digested with ApaI (Promega, Southampton, UK) according to Miranda et al. (1996). DNA fragments were separated on 1%, w/v agarose gels in 0.5× TBE buffer at 14°C using a CHEF DRII apparatus (Bio-Rad) with 6 V/cm, pulsed from 5 to 35 s for 24 h. Gels were stained with ethidium bromide and were scanned using the Bio-Rad Gel Doc software image capturing system. The Dice coefficient was used to calculate similarities, and the unweighted pair group method using average linkages (UPGMA) was used for cluster analysis with BioNumerics software v. 7.1 (Applied Maths, St Martens-Latem, Belgium). Isolates that clustered together with a similarity of >85% were considered to belong to the same PFGE clone (Towner et al., 2008; McCracken et al., 2011).

## Results

### Antimicrobial Resistance

**FIGURE 1:**
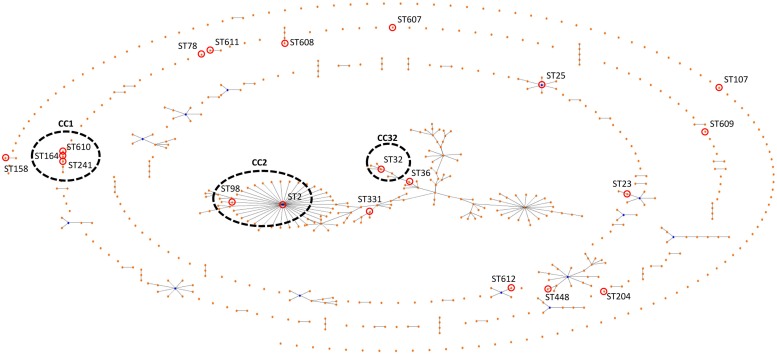
**eBURST figure comparing the data of the tested isolates with all of the database.** Assigned numbers for the new STs, ST-607, ST-608, ST-609, ST-610, ST-611, and ST-612. The corresponding sequence types are shown beside each isolate number. CCs assigned by eBURST analysis as indicated.

Of the 100 isolates tested 33 (33%) were resistant to at least three or more classes of antibiotic (**Table 1**). The majority of isolates were collected from urine samples (33%) followed by wound samples (18%; **Figure 1**). Only isolate KO-116 conferred reduced sensitivity to colistin (MIC 2.5 mg/L) as well as all other types of antibiotics tested. KO-116 was isolated from a 62 years-old male patient admitted to the ICU ward.

### Detection and Identification of *bla*_OXA_ Genes

All the 100 isolates contained a *bla*_OXA-51_-like gene. The amplified product of the *bla*_OXA-51_-like gene for one isolate (KO-12) recovered from blood of a patient with chronic renal failure did not show the expected product size of 353 bp. Sequencing the *bla*_OXA-51_-like variant showed the insertion of ISAba19 at position 380 in *bla*_OXA-78_. KO-12 contained *bla*_OXA-24_ and had an intermediate resistance to ciprofloxacin and was sensitive to carbapenems, colistin, and tigecycline. Therefore since this isolate did not match our MDR criteria it was not included in this study.

Of the 33 MDR *A. baumannii* isolates, 28 (85%) contained *bla*_OXA-23_ and 2 (6%) *bla*_OXA-24_. We did not detect *bla*_OXA-58_ genes in any of the tested isolates. *bla*_VIM_, *bla*_IMP_, *bla*_GES_ and *bla*_NDM_, *bla*_VEB_ genes were not identified. All the 33 isolates contained a *bla*_ADC-like_ gene.

### Detection and Identification of *bla*_PER-1_

In three out of six *bla*_PER-1_ positive isolates (KO-5, 22, and 31) the genetic environment of *bla*_PER-1_ consisted of two copies of ISPa12 (tnpiA1) surrounding the *bla*_PER-1_ gene with a spacer region of 154 bp in both extremes. *bla*_PER-1_ was located on a plasmid of ca. 140-kb. The plasmid was not deleted after 30 days of curing and by PCR the *bla*_PER-1_ gene was detected indicating that the plasmid was highly stable. The novel transposon has the accession number: KF978125. As for the other three cephalosporine resistant (ceftazidime MIC >256 mg/l) *bla*_PER-1_ positive isolates (KO-4, 34, and 82) we did not detect ISPa12 upstream the gene.

### Characterization of *A. baumannii* Isolates by MLST

Multilocus-sequence typing analysis of the 33 *A. baumannii* isolates identified 20 different STs. Of the 20 STs determined by Institut Pasteur scheme, six were novel. They included ST-607, ST-608, ST-609, ST-610, ST-611, and ST-612. The predominant ST was ST-2, which was comprised of six isolates (18%), followed by ST-25 and ST-32, both with four isolates (12%). Other less common STs were ST-78 with two isolates (6%), ST-158 also comprising of two isolates (9%) and 15 STs were presented only once in the data set (**Figure 1**). The isolates from urine samples were the most diverse belonging to nine different STs. Of the 11 urine isolates, (18% [*n* = 2]) were ST-2, (18% [*n* = 2]) were ST-25 while the other seven isolates were of other STs.

### eBURST Analysis

By means of the eBURST algorithm, the 33 isolates were clustered into at least four CCs and 11 singletons (**Figure 1**). The largest CC, CC2 comprised 18% (*n* = 7) of the isolates, while 23.3% of the isolates belonged to ST-2 (*n* = 6) or ST-98 (*n* = 1).

### Pulsed-Field Gel Electrophoresis

**FIGURE 2:**
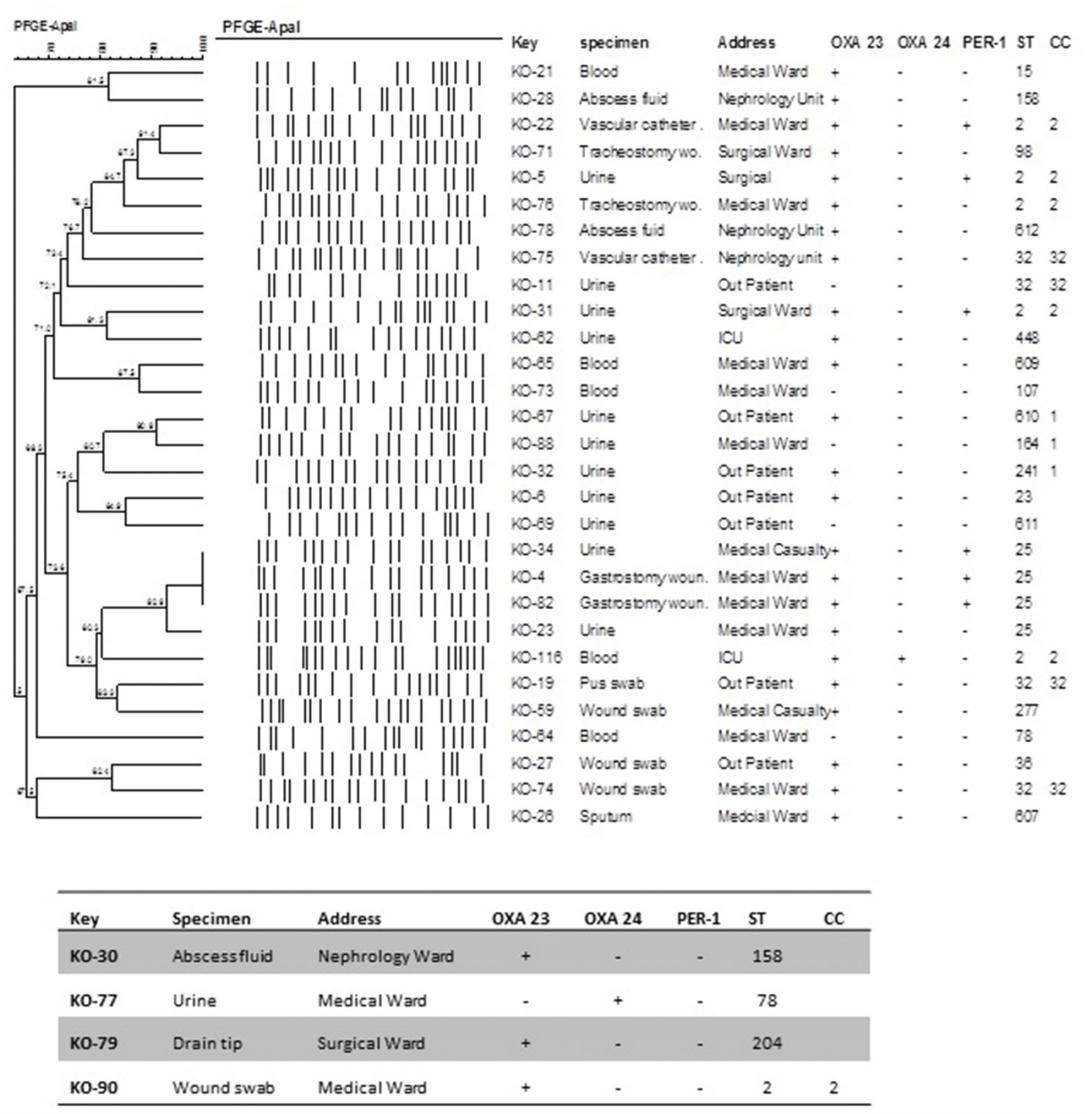
**Dendogram illustrating the PFGE patterns of *A. baumannii* isolates after restriction with ApaI enzyme.** PFGE settings: Similarity coefficient Dice. Optimization: 1.5%, Tolerance: 1.5%, Tolerance change: 1%, Uncertain bands: Ignore, Relaxed doublet matching. Clustering method: UPGMA. Active zones: [0.0–100.0%].

Pulsed-field gel electrophoresis analysis suggested the presence of five major clusters with two or more isolates (**Figure 2**). Only three isolates displayed identical PFGE patterns. Interestingly they were ST25 containing the *bla*_PER-1_ gene (KO 4, 34, 82) indicating the possibility of a pseudo outbreak. From the dendogram we can conclude that not all the isolates belonging to the same ST necessarily shared identical PFGE pattern. However, none of the identical PFGE patterns were from different STs. In addition the presence or absence of *bla*_OXA-23_ is neither associated with the PFGE patterns nor with STs. We can also observe that in most cases the CC correlated with the PFGE lineages. Four of the isolates were not typeable by PFGE.

## Discussion

In the current study we report on the diversity of the clinical MDR *A. baumannii* isolated from one of the largest governmental hospitals in Kuwait. MDR isolates pose a great risk to the treatment of patients in Al-Amiri hospital. Following the identification of the isolates by molecular methods, the amplified *bla*_OXA-51_-like gene product of one isolate (KO-12) recovered from blood showed the insertion of the ISAba19 at position 379 in *bla*_OXA-78_ similarly to a previous report (Zander et al., 2013). As suggested, failure to detect the correct *bla*_OXA-51_-like product should not be a sufficient reason to exclude the isolate as *A. baumannii*. However, since this isolate could not be classified as MDR (resistance to antibiotics in more than three antimicrobial categories), it was therefore excluded from this study.

We employed PFGE and MLST to type the isolates as both techniques have high discriminatory powers. MLST is suitable to trace the evolutionary history of *A. baumannii* and to build population genetic structure by separating epidemiologically unrelated isolates (Bartual et al., 2005) and PFGE typing is a useful epidemiological tool for outbreak analysis. According to our findings the 33 MDR *A. baumannii* comprised of 20 different STs and at least three CCs of which six novel STs were identified. Noticeably emerging STs (Villalón et al., 2011; Giannouli et al., 2013) such as ST15, ST78, and ST25 were detected. The most common ST was ST-2 and CC2 was the largest CC. The detection of six novel STs supports the notion that *A. baumannii* is diverse in clonal origins and/or is undergoing clonal expansion continuously. Due to the close proximity of Kuwait to other countries in the Gulf region, it is not surprising to detect common STs: ST-2 and ST-25 have been previously identified in Saudi Arabia (Aly et al., 2014) and in Yemen (Bakour et al., 2014). Moreover; ST-2 is known as an endemic strain in European countries including Italy and Spain (Villalón et al., 2011; Mezzatesta et al., 2014). We also detected the novel international CC32 with known epidemic potential (Da Silva et al., 2014). It has been shown that strains assigned to sequence types ST2, ST25, and ST78 produce biofilm more efficiently and are able to form a robust biofilm pellicle at the air-liquid interface of the culture medium (Giannouli et al., 2013).

Our study group is too small to find statistical correlation between either the STs and ward to suggest endemicity or between the sample type and the STs. The most alarming aspect in the ICU was the isolation of KO-116 belonging to ST-2 with reduced sensitivity to colistin (MIC = 2.5 mg/L) which is known to have the potential to become endemic and pose a greater risk to treating infected patients (Giannouli et al., 2013). In this study we also detected ST15 for the first time in the Middle East. According Diancourt et al. (2010), the rapid clonal expansion of ST15 and its evolutionary success is due to the fact that almost all *A. baumannii* belonging to ST15 are MDR.

The *bla*_OXA-23_ gene is considered a virulence biomarker and a significant cause of carbapenem resistance worldwide (Luo et al., 2015) and is either located on the chromosome or on plasmids. Transposons Tn2006, Tn2007, and Tn2008 have been identified to contain *bla*_OXA-23_ (Mugnier et al., 2010). In our study *bla*_OXA-23_ was also the most common oxacillinase gene identified, similar to other Gulf countries (Zowawi et al., 2015). Our results show that there was a strong correlation between the presence of *bla*_OXA-23_ and MDR phenotype. However, the presence of this gene was not associated with STs indicating that *bla*_OXA-23_ is most likely present on a mobile element. In contrast to previous reports on the prevalence of *bla*_OXA-58_, *bla*_GES_ *bla*_IMP_, and *bla*_VIM_ in *A. baumannii* (Al-Sweih et al., 2012; Bonnin et al., 2013; Zowawi et al., 2015), we did not detect any of the mentioned resistance genes among the 33 MDR isolates. These variations might be attributed to differences in antibiotic treatment strategies at hospitals which in turn may influence the evolutionary direction of *A. baumannii*.

Another interesting finding was a novel genetic transposon-like structure related to *bla*_PER-1_ in *A. baumannii*. Three isolates (KO-5, KO-22, and KO-31) were highly resistant to ceftazidime (MIC >256 mg/l). The PFGE patterns of the three isolates were not identical, however, they belonged to ST2. In these isolates the ISAba1 element was detected upstream the *bla*_ADC-like_ gene, which has been described as the most common mechanism of ceftazidime resistance (Peleg et al., 2008). Nevertheless, *bla*_ADC-like_ gene was not the exclusive mechanism of resistance to ceftazidime. The second mechanism was the presence of ESBL *bla*_PER-1_ detected on a plasmid of ca. 140-kb. Previously The *bla*_PER-1_ gene has been associated with ISPa12 upstream, which increases its expression, and bracketed downstream by ISPa13, comprising the transposon Tn1213 (Poirel et al., 2011). However, in this study the *bla*_PER-1_ gene was surrounded by two copies of ISPa12 (TnpA1) representing a novel genetic structure harboring this enzyme. The genetic environment of *bla*_PER-1_ was composed by two copies of ISPa12, flanking either side of the ESBL gene with a spacer region of 154 bp at both extremities (accession no. KF978125). The ISPa12 element was originally identified in *Pseudomonas aeruginosa* species, which may suggest that the *bla*_PER-1_ gene may have been acquired from this microorganism. The unsuccessful plasmid curing experiments implied that the large 140-kb plasmid is highly stable. Nevertheless the presence of two copies of ISPa12 confirms the remarkable ability of *A. baumannii* to utilize insertion sequences to increase the expression of resistance genes. This characteristic may be a contributing factor to instigate *A. baumannii* as a threatening nosocomial pathogen. PFGE analysis revealed that the presence of the *bla*_PER-1_ gene is not limited to a certain pattern which correlates with the MLST results.

PFGE showed that within the time line of our research different genotypes of MDR *A. baumannii* circulated in the hospital. Only three of the isolates containing the *bla*_PER-1_ gene showed identical PFGE patterns which leads us to believe there is a possibility of a pseudo outbreak.

In Kuwait cephalosporins are widely used to treat infections; therefore it is important to monitor and to control the spread of horizontal transfer by administering the correct antibiotic and preventing the spread of resistant strains among hospitalized patients.

## Conclusion

While PFGE profiles did not always correlate with the MLST, nevertheless both techniques defined the MDR isolates as multi-clonal circulating in hospital ward simultaneously. The outcome of our study provides a baseline for future longitudinal research whereby transitory and endemic strains can be identified.

## Author Contributions

LV, AO-C, AD, KAO conceived and designed the study. LV, KD, AO-C, KAO acquired the data. LV, KD, AFOC, KO, BAE analysed the data. LV, AFOC, AAD, BAE drafted and critically evaluated the manuscript. All authors approved the final version of the manuscript.

## Conflict of Interest Statement

The authors declare that the research was conducted in the absence of any commercial or financial relationships that could be construed as a potential conflict of interest.

## Footnotes

1 www.ncbi.nlm.nih.gov

2 http://www.pasteur.fr/mlst

3 http://eburst.mlst.net/v3/enter_data/single/
