# Supplementary material for: Diversity of multi-drug resistant Acinetobacter baumannii population in a major hospital in Kuwait
Source: Front Microbiol. 2015 Jul 23;6:743. doi: 10.3389/fmicb.2015.00743 (PMC4513246; doi:10.3389/fmicb.2015.00743)
Supplement: Supplementary file 1 [file Presentation_1.PDF]

Detection of $\textit{bla}_{\text{OXA-51-like}}$

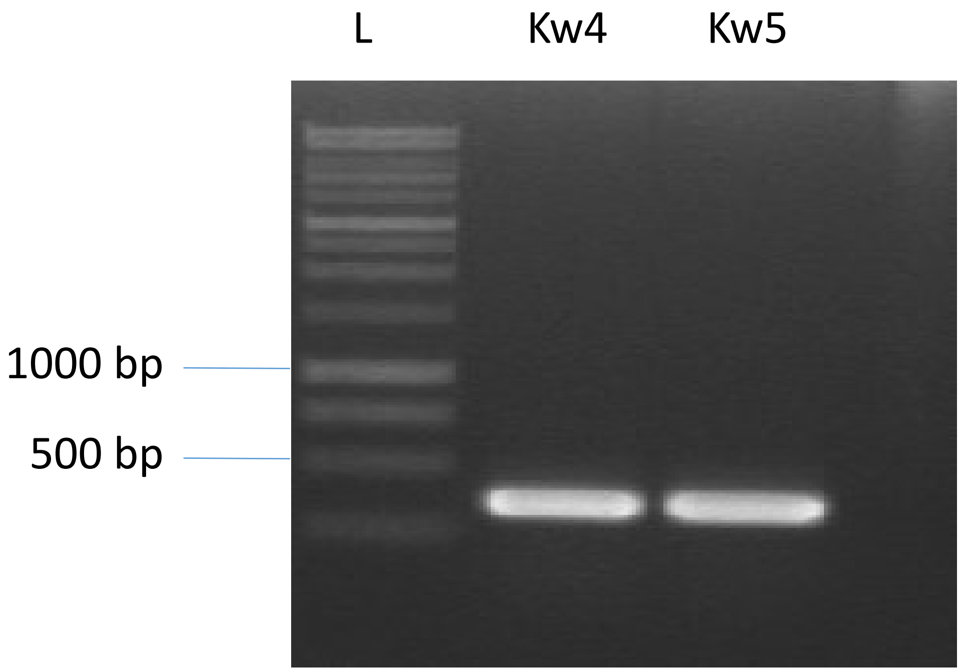

Expected size: 353 bp
(Woodford *et al.* 2006)

Detection of $\textit{bla}_{\text{PER-like}}$

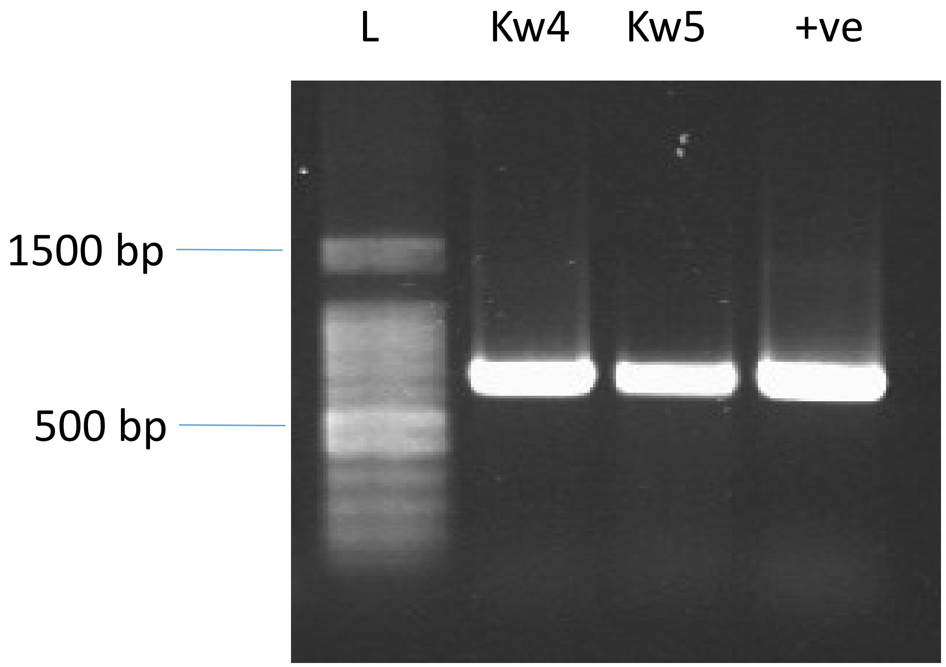

Expected size: 715 bp
Ta: 57°C

Detection of *bla*$_{ADC\text{-}like}$

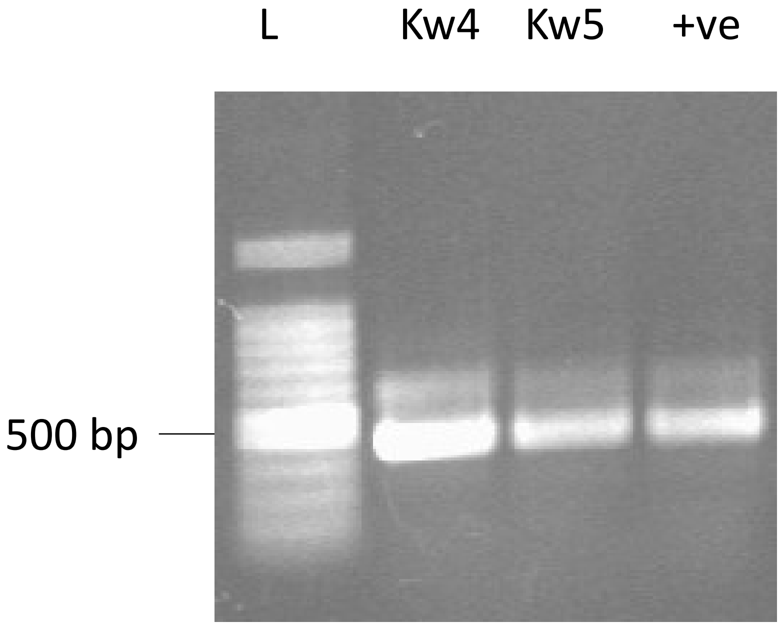

Expected size: 420 bp
(Ruiz *et al.* 2007)

Upstream of *bla*$_{ADC\text{-}like}$

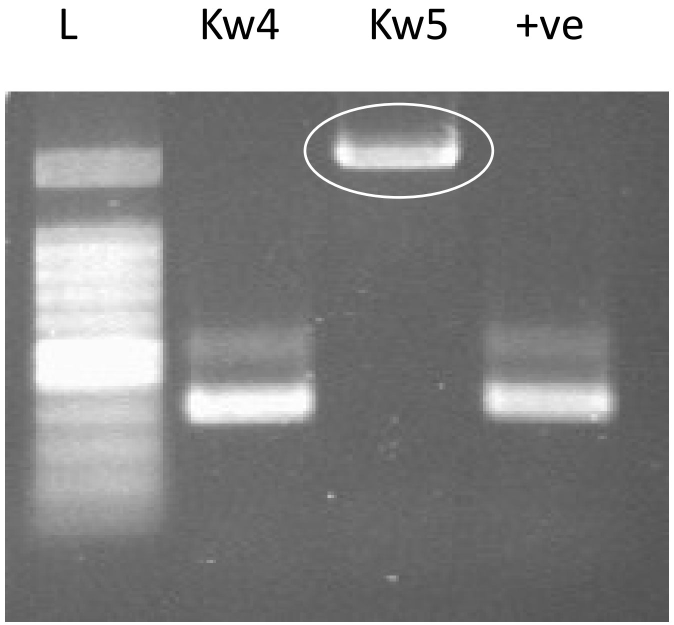

Expected size (no IS): 360 bp
(Lopes, 2012)
Ta: 57°, 35 cycles.

RESULT: IS*Aba1*

Detection of ORF-513

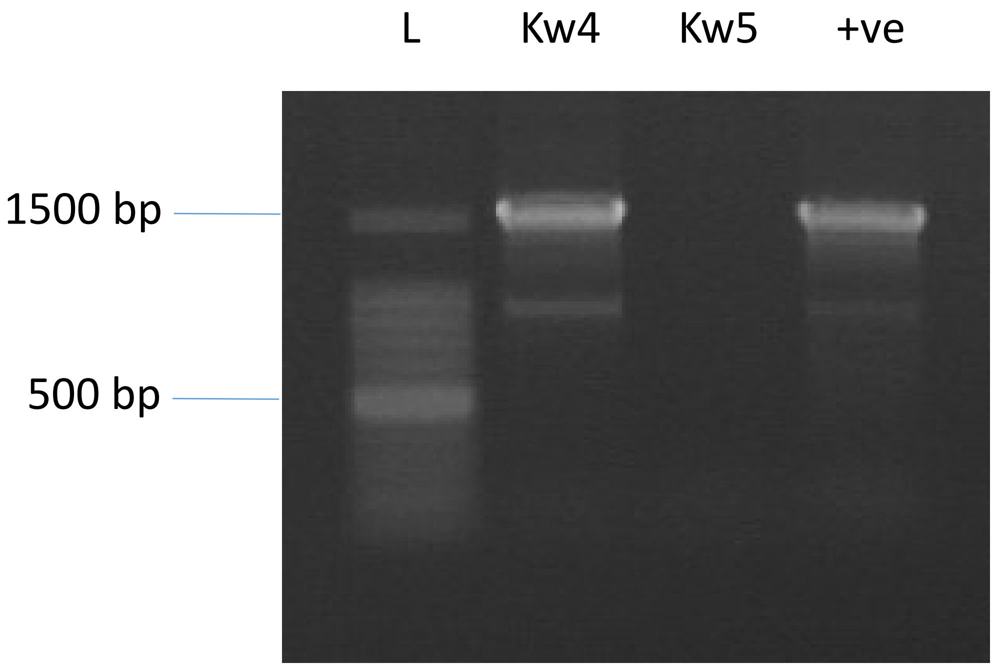

Expected size: 1520 bp
Ta: 57°C
*30 cycles

Detection PER-ORF513

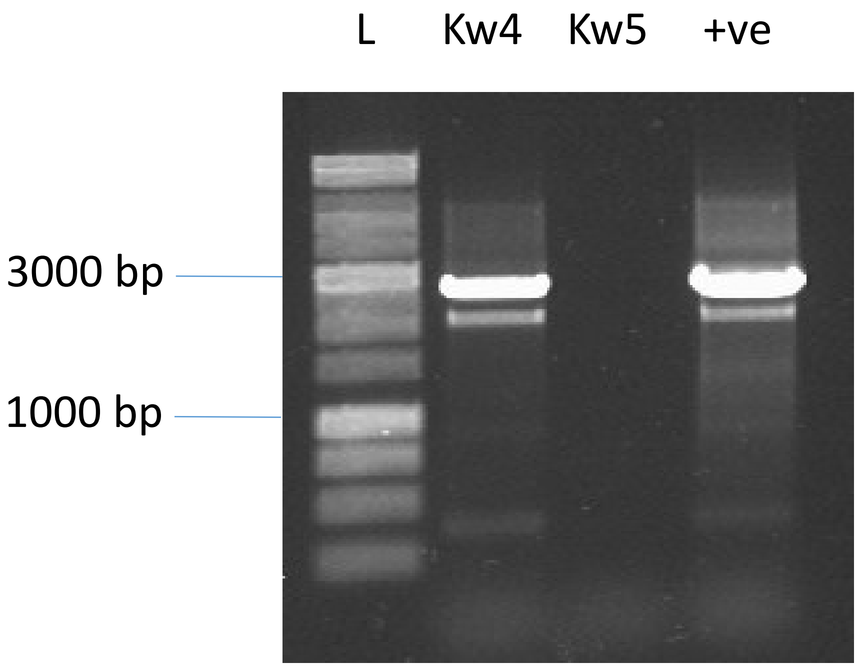

Expected size: 2235 bp
Ta: 55°C

Inverse PCR Kw4 (EcoRI) → Downstream *bla*$_{\text{PER-like}}$

Download | GenBank | Graphics | ▼ Next ▲ Previous Descriptions

Acinetobacter baumannii strain NF161710 retrotransposon ISCR1 putative transposase gene, partial cds, and extended-spectrum beta-lactamase PER-1 (blaPER-1), putative glutathione-S-transferase (gst), and putative ATP-binding/permease protein genes, complete cds

Sequence ID: gb|JQ780836.1| **Length:** 6761 **Number of Matches:** 1

Related Information

Range 1: 2585 to 2963 GenBank Graphics ▼ Next Match ▲ Previous Match

Score	Expect	Identities	Gaps	Strand
701 bits(379)	0.0	379/379(100%)	0/379(0%)	Plus/Plus

```
Query  9     CAAATTAACAGACTATCAGCACTATCTAAGCCGCTGACTCTGGTTGTACACTAAAGCTAT  68
             ||||||||||||||||||||||||||||||||||||||||||||||||||||||||||||
Sbjct  2585  CAAATTAACAGACTATCAGCACTATCTAAGCCGCTGACTCTGGTTGTACACTAAAGCTAT  2644

Query  69    GGTTTGAAATTGGAGTAGGTTATGCAGTTATTAGGTTCAGTGGCTTCCCCTTTTGTTCGT  128
             ||||||||||||||||||||||||||||||||||||||||||||||||||||||||||||
Sbjct  2645  GGTTTGAAATTGGAGTAGGTTATGCAGTTATTAGGTTCAGTGGCTTCCCCTTTTGTTCGT  2704

Query  129   CGTTTACGTTTAGTACTGGCAGGGCAACCTTATCAGTTTGTAGCGCTTAATATTTTTGAG  188
             ||||||||||||||||||||||||||||||||||||||||||||||||||||||||||||
Sbjct  2705  CGTTTACGTTTAGTACTGGCAGGGCAACCTTATCAGTTTGTAGCGCTTAATATTTTTGAG  2764

Query  189   TCTGAAGGCCGTTCAGTGTTGGTACAACATAATCCGGCACGCAAAGTGCCTGTGTTAGTG  248
             ||||||||||||||||||||||||||||||||||||||||||||||||||||||||||||
Sbjct  2765  TCTGAAGGCCGTTCAGTGTTGGTACAACATAATCCGGCACGCAAAGTGCCTGTGTTAGTG  2824

Query  249   GATGGAGAGCAGGTTATTTTTGATTCAGGCGTAATTTATCGTTATTTGGCTTCGAAACTG  308
             ||||||||||||||||||||||||||||||||||||||||||||||||||||||||||||
Sbjct  2825  GATGGAGAGCAGGTTATTTTTGATTCAGGCGTAATTTATCGTTATTTGGCTTCGAAACTG  2884

Query  309   AAATTCAAACCATTGAGCTGGGATCAGGAAAACGGTCTGACGACCATCAATGCCTGCACA  368
             ||||||||||||||||||||||||||||||||||||||||||||||||||||||||||||
Sbjct  2885  AAATTCAAACCATTGAGCTGGGATCAGGAAAACGGTCTGACGACCATCAATGCCTGCACA  2944

Query  369   GACTCCTTGGTTGAATTAC  387
             |||||||||||||||||||
Sbjct  2945  GACTCCTTGGTTGAATTAC  2963
```

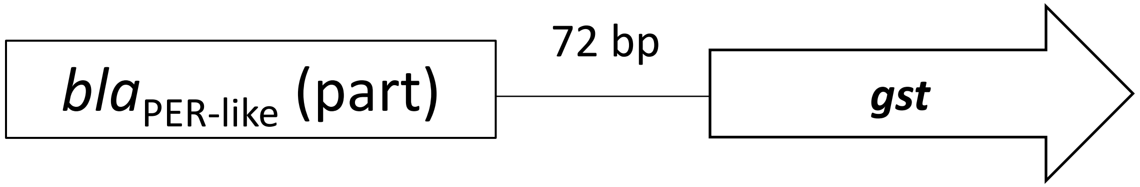

Upstream *bla*$_{\text{PER-like}}$ Kw4

IntI1

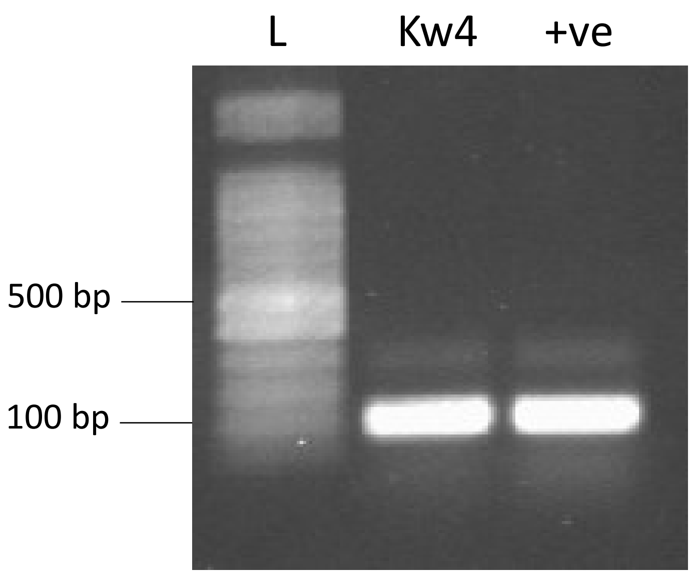

- IntI1-F: 5’-CAGTGGACATAAGCCTGTTC-3’
- IntI1-R: 5’-CCCGAGGCATAGACTGTA-3’
- Ta: 57°C
- Amplicon size: 160 bp

3’CS

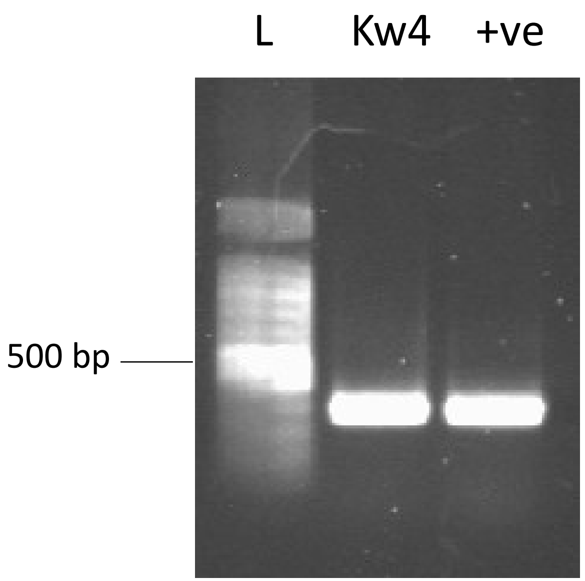

- [Sul1-mF – 3CS-R]
- Ta: 57°C
- Amplicon size: 347 bp

IntI1-3’CS

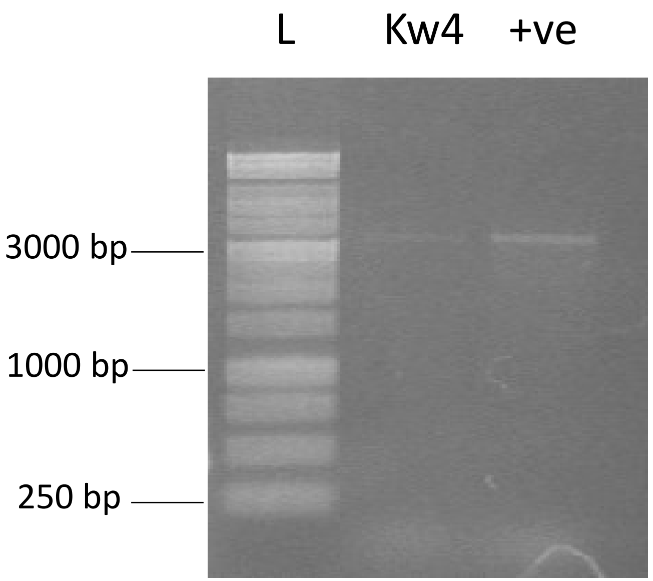

- [IntI1-F – 3CS-R]
- Ta: 57°C
- Amplicon size: ~ 2800 bp

Upstream $bla_{\text{PER-like}}$ Kw4

Sul1-ORF513

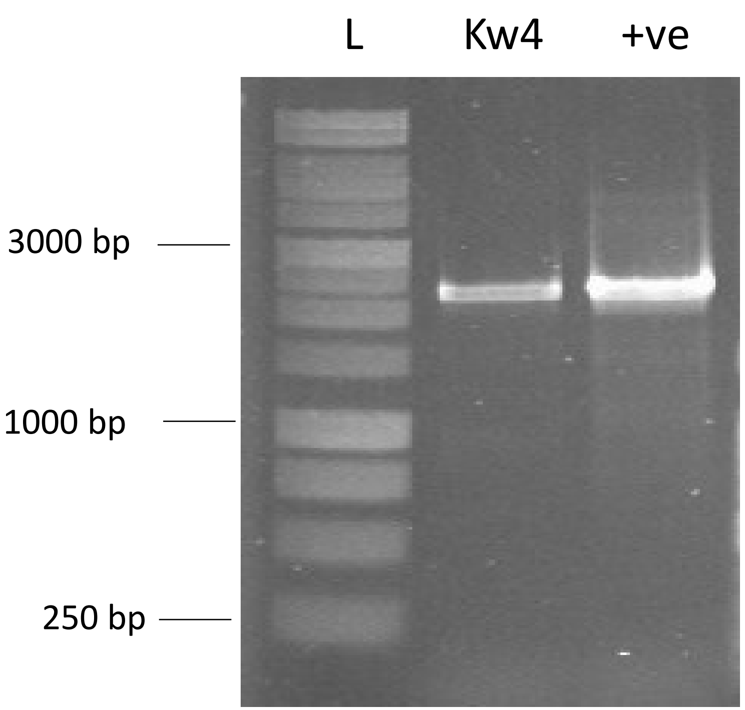

- [Sulm-1F – ORF513-R]
- Ta: 57°C
- Amplicon size: ~ 2800 bp

ORF513-PER-R

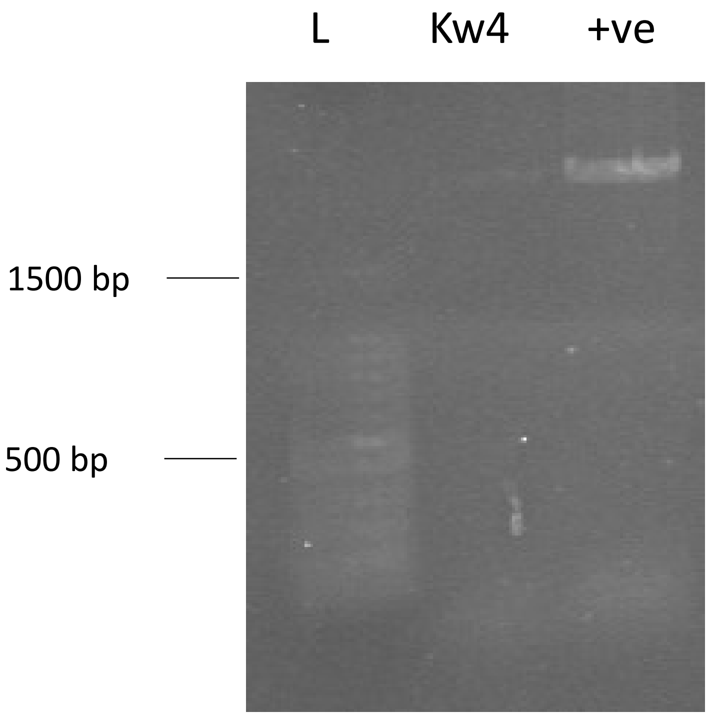

- [ORF513-F – PER-R]
- Ta: 57°C
- Amplicon size: > 1500 bp

Inverse PCR Kw5 (EcoRI) → Downstream *bla*$_{\text{PER-like}}$

Download ⌄ GenBank Graphics Sort by: E value

Acinetobacter baumannii 1656-2, complete genome

Sequence ID: gb|CP001921.1| **Length:** 3940614 **Number of Matches:** 2

Range 1: 285733 to 286226 GenBank Graphics ▼ Next Match ▲ Previous Match

Score	Expect	Identities	Gaps	Strand
902 bits(488)	0.0	492/494(99%)	0/494(0%)	Plus/Minus

Features: Extended-spectrum beta-lactamase PER-1 precursor
Transposase TnpA1

```
Query  1       CTTGACGCATAAATTCAGGAGTTCCGAGCTTAACTAACTGATTATGGAATGGTTTAAGGC  60
               ||||||||||||||||||||||||||||||||||||||||||||||||||||||||||||
Sbjct  286226  CTTGACGCATAAATTCAGGAGTTCCGAGCTTAACTAACTGATTATGGAATGGTTTAAGGC  286167

Query  61      GTACTGACATCGAATGCAAGTGGTTAAAGTAACGATGTATATCAGCGATTGTGCGGGCAT  120
               ||||||||||||||||||||||||||||||||||||||||||||||||||||||||||||
Sbjct  286166  GTACTGACATCGAATGCAAGTGGTTAAAGTAACGATGTATATCAGCGATTGTGCGGGCAT  286107

Query  121     TTCCACTTCCCAGGGCACACACAAATGCCAGGCAAAGTTTTGCGGGAGTGATCAGGCGAA  180
               ||||||||||||||||||||||||||||||||||||||||||||||||||||||||||||
Sbjct  286106  TTCCACTTCCCAGGGCACACACAAATGCCAGGCAAAGTTTTGCGGGAGTGATCAGGCGAA  286047

Query  181     AGCGTTCAATCATGTGAGTTTGAACACCAAGTGAATTTAATTGTTGCTCTGAAAACAGGG  240
               ||||||||||||||||||||||||||||||||||||||||||||||||||||||||||||
Sbjct  286046  AGCGTTCAATCATGTGAGTTTGAACACCAAGTGAATTTAATTGTTGCTCTGAAAACAGGG  285987

Query  241     CTTCAAAAATGGTTGAAAATGCGGTAATCTGATTTTGCTTCATTCGTTTTAGCCCTCTGG  300
               ||||||||||||||||||||||||||||||||||||||||||||||||||||||||||||
Sbjct  285986  CTTCAAAAATGGTTGAAAATGCGGTAATCTGATTTTGCTTCATTCGTTTTAGCCCTCTGG  285927

Query  301     GCGTTCTATTTTATTCGCAAAATCAATTAGATCACGAATGAAGCACCTATTCAAATCCTA  360
               ||||||||||||||||||||||||||||||||||||||||||||||||||||||||||||
Sbjct  285926  GCGTTCTATTTTATTCGCAAAATCAATTAGATCACGAATGAAGCACCTATTCAAATCCTA  285867

Query  361     AAGATCATACGTATGAAAAGGACAATCCGATGAATGTCATTATAAAAGCTGTAGTTACTG  420
               ||||||||||||||||||||||||||||||||||||||||||||||||||||||||||||
Sbjct  285866  AAGATCATACGTATGAAAAGGACAATCCGATGAATGTCATTATAAAAGCTGTAGTTACTG  285807

Query  421     CCTCGACGCTACTGATGGTATCTTTTAGTTCATTCGAAACCTCAGCGCAATCCCCACTGT  480
               ||||||||||||||||||||||||||||||||||||||||||||||||||||||||||||
Sbjct  285806  CCTCGACGCTACTGATGGTATCTTTTAGTTCATTCGAAACCTCAGCGCAATCCCCACTGT  285747

Query  481     TAAAGAAGCAAATT  494
               ||||  ||||||||
Sbjct  285746  TAAAAGAGCAAATT  285733
```

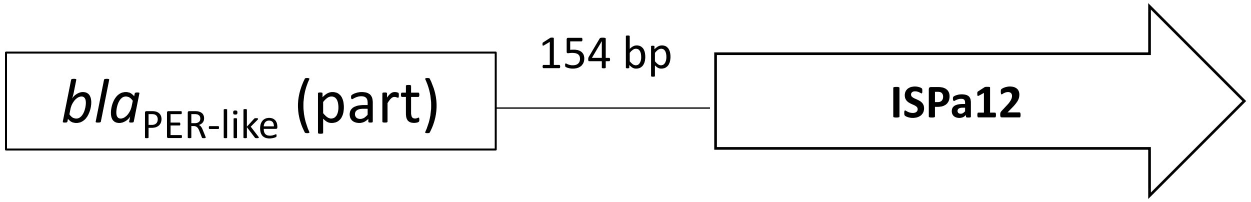

Inverse PCR Kw5 (EcoRI) → Upstream *bla*$_{\text{PER-like}}$

Acinetobacter baumannii 1656-2, complete genome

Sequence ID: gb|CP001921.1| **Length:** 3940614 **Number of Matches:** 2

Range 1: 285782 to 286404 GenBank Graphics ▼ Next Match ▲ Previous Match

Score	Expect	Identities	Gaps	Strand
1129 bits(611)	0.0	620/624(99%)	2/624(0%)	Plus/Plus

Features: Extended-spectrum beta-lactamase PER-1 precursor
Transposase TnpA1

```
Query  2       AAAGATACCATCAGTAGCGTCGAGGCAGTAACTACAGCTTTTATAATGACATTCATCGGA  61
               ||||||||||||||||||||||||||||||||||||||||||||||||||||||||||||
Sbjct  285782  AAAGATACCATCAGTAGCGTCGAGGCAGTAACTACAGCTTTTATAATGACATTCATCGGA  285841

Query  62      TTGTCCTTTTCATACGTATGATCTTTAGGATTTGAATAGGTGCTTCATTCGTGATCTAAT  121
               ||||||||||||||||||||||||||||||||||||||||||||||||||||||||||||
Sbjct  285842  TTGTCCTTTTCATACGTATGATCTTTAGGATTTGAATAGGTGCTTCATTCGTGATCTAAT  285901

Query  122     TGATTTTGCGAATAAAATAGAACGCCCAGAGGGCTAAAACGAATGAAGCAAAATCAGATT  181
               ||||||||||||||||||||||||||||||||||||||||||||||||||||||||||||
Sbjct  285902  TGATTTTGCGAATAAAATAGAACGCCCAGAGGGCTAAAACGAATGAAGCAAAATCAGATT  285961

Query  182     ACCGCATTTTCAACCATTTTTGAAGCCCTGTTTTCAGAGCAACAATTAAATTCACTTGGT  241
               ||||||||||||||||||||||||||||||||||||||||||||||||||||||||||||
Sbjct  285962  ACCGCATTTTCAACCATTTTTGAAGCCCTGTTTTCAGAGCAACAATTAAATTCACTTGGT  286021

Query  242     GTTCAAACTCACATGATTGAACGCTTTCGCCTGATCACTCCCGCAAAACTTTGCCTGGCA  301
               ||||||||||||||||||||||||||||||||||||||||||||||||||||||||||||
Sbjct  286022  GTTCAAACTCACATGATTGAACGCTTTCGCCTGATCACTCCCGCAAAACTTTGCCTGGCA  286081

Query  302     TTTGTGTGTGCCCTGGGAAGTGGAAATGCCCGCACAATCGCTGATATACATCGTTACTTT  361
               ||||||||||||||||||||||||||||||||||||||||||||||||||||||||||||
Sbjct  286082  TTTGTGTGTGCCCTGGGAAGTGGAAATGCCCGCACAATCGCTGATATACATCGTTACTTT  286141

Query  362     AACCACTTGCATTCGATGTCAGTACGCCTTAAACCATTCCATAATCAGTTAGTTAAGCTC  421
               ||||||||||||||||||||||||||||||||||||||||||||||||||||||||||||
Sbjct  286142  AACCACTTGCATTCGATGTCAGTACGCCTTAAACCATTCCATAATCAGTTAGTTAAGCTC  286201

Query  422     GGAACTCCTGAATTTATGCGTCAAGTGTTTGAGCAGGCGCTGGCACTTCATTTGCCAGCC  481
               ||||||||||||||||||||||||||||||||||||||||||||||||||||||||||||
Sbjct  286202  GGAACTCCTGAATTTATGCGTCAAGTGTTTGAGCAGGCGCTGGCACTTCATTTGCCAGCC  286261

Query  482     ATGCACACCTTTTCGGACGCCTATCGGGGGCATTTCAAGCAGGTACTGTTGCAGGATGGC  541
               ||||||||||||||||||||||||||||||||||||||||||||||||||||||||||||
Sbjct  286262  ATGCACACCTTTTCGGACGCCTATCGGGGGCATTTCAAGCAGGTACTGTTGCAGGATGGC  286321

Query  542     ACCAGTTTCGCTGTTCATGACGGCCTCAGCCTGCATTTCCCCGGGCGATTCAGTACACAC  601
               ||||||||||||||||||||||||||||||||||||||||||||||||||||||||||||
Sbjct  286322  ACCAGTTTCGCTGTTCATGACGGCCTCAGCCTGCATTTCCCCGGGCGATTCAGTACACAC  286381

[illegible]
```

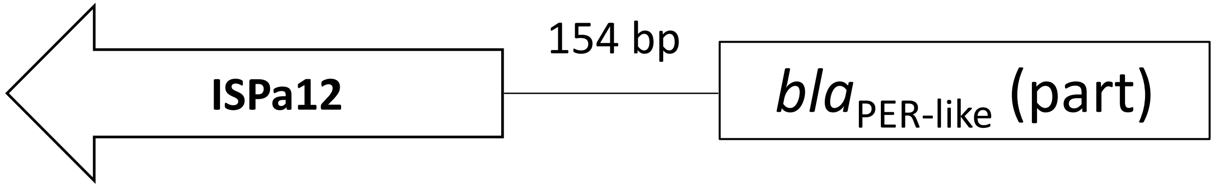

Plasmid Profile Kuwait samples

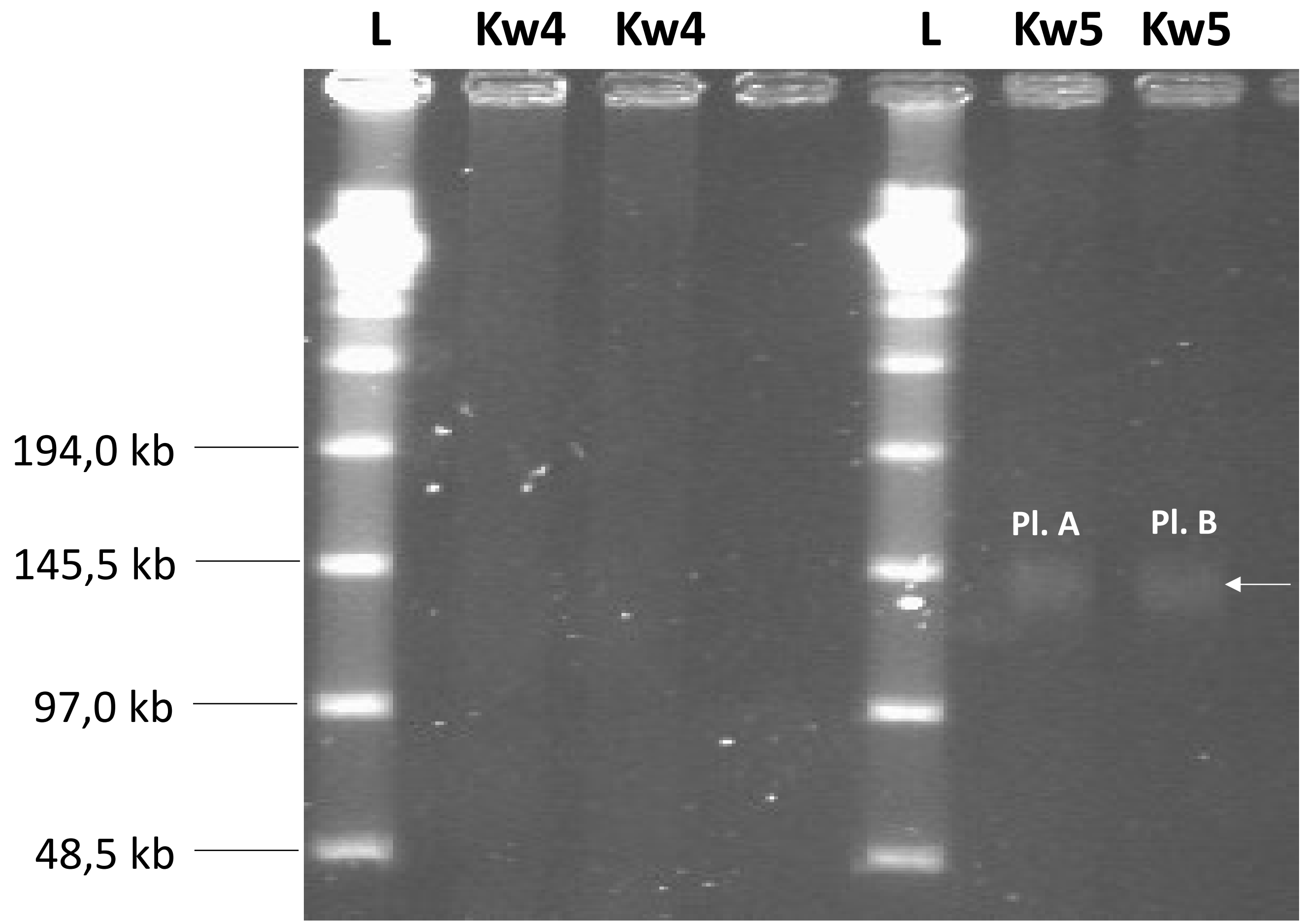

PFGE: 6 V/cm; 20 h; initial pulse 5s; final pulse 20s

Detection of *bla*$_{\text{PER-like}}$ in plasmid

L Pl. A Pl. B DNAt

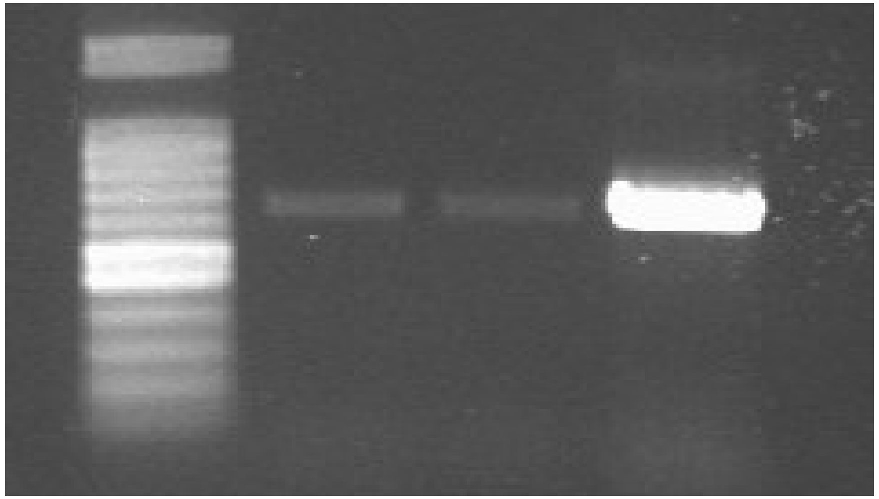

Ta: 57°
Primers: [PER-F – PER-R]

Detection of *16S*

L Pl. A Pl. B DNAt

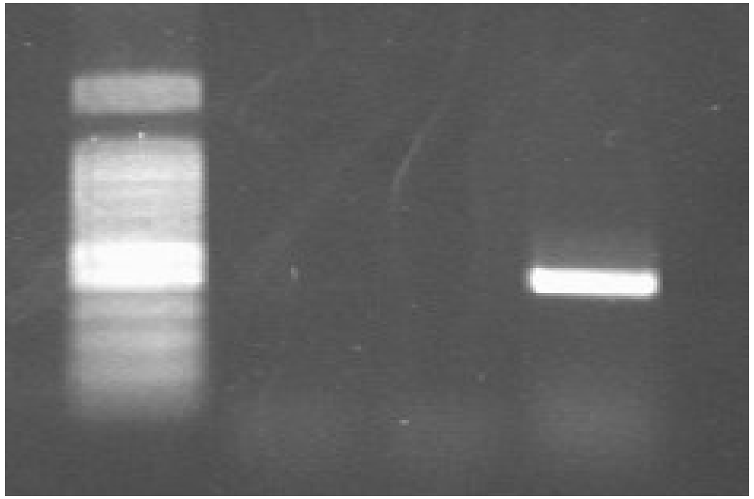

Ta: 55°
Primers: [16S-F – 16S-R]
Expected size: 426 bp
***20 cycles**

Plasmid Curing Kw5
Incubating at 45°C in MacConkey agar

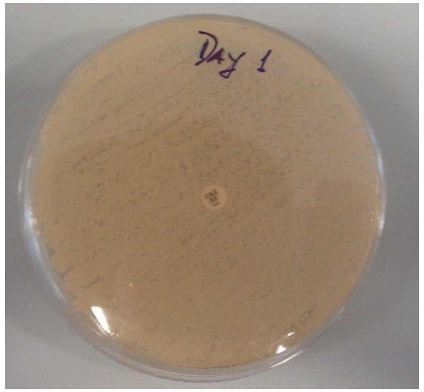

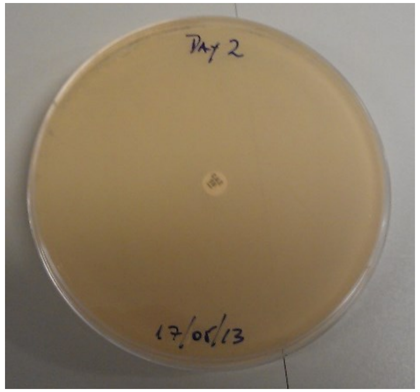

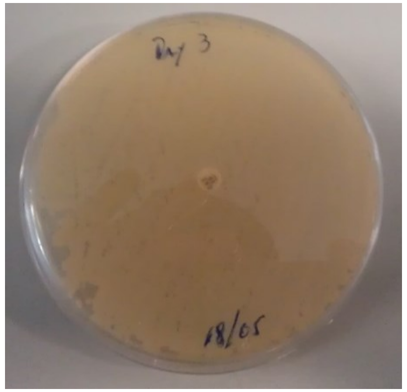

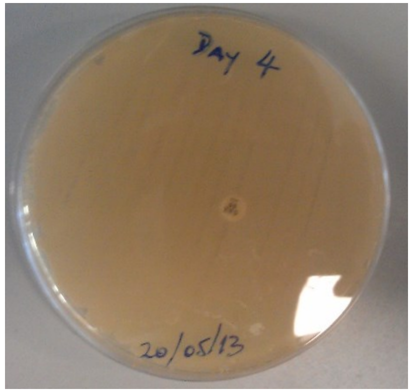

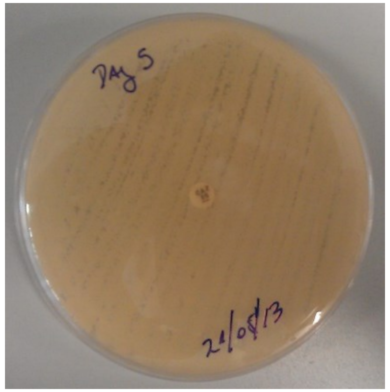

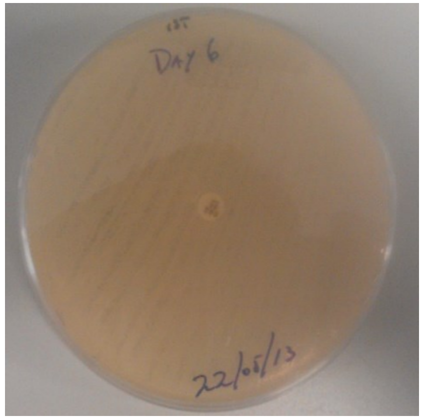

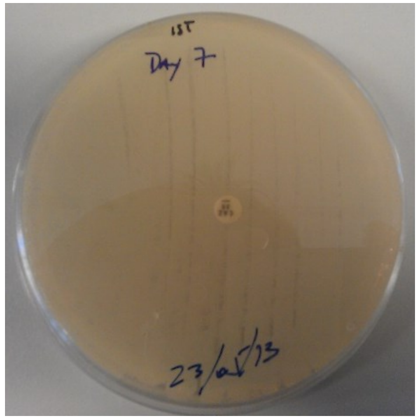

PER-like detection by PCR

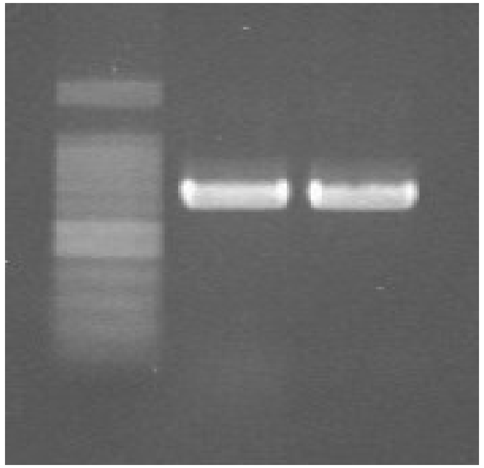

11 Days incubating at 42°C → PER-like (+)
